# Elongated Membrane Tethers, Individually Anchored by High Affinity α_4_β_1_/VCAM-1 Complexes, Are the Quantal Units of Monocyte Arrests

**DOI:** 10.1371/journal.pone.0064187

**Published:** 2013-05-17

**Authors:** Calvin Chu, Emrah Celik, Felix Rico, Vincent T. Moy

**Affiliations:** Department of Physiology and Biophysics, University of Miami Miller School of Medicine, Miami, Florida, United States of America; Swiss Federal Institute of Technology Zurich, Switzerland

## Abstract

The α_4_β_1_ integrin facilitates both monocyte rolling and adhesion to the vascular endothelium and is physiologically activated by monocyte chemoattractant protein (MCP-1). The current study investigated the initial events in the adhesion of THP-1 cells to immobilized Vascular Cell Adhesion Molecule 1 (VCAM-1). Using AFM force measurements, cell adhesion was shown to be mediated by two populations of α_4_β_1_/VCAM-1 complexes. A low affinity form of α_4_β_1_ was anchored to the elastic elements of the cytoskeleton, while a higher affinity conformer was coupled to the viscous elements of the cell membrane. Within 100 ms of contact, THP-1 cells, stimulated by co-immobilized MCP-1, exhibited a tremendous increase in adhesion to VCAM-1. Enhanced cell adhesion was accompanied by a local decoupling of the cell membrane from the cytoskeleton and the formation of long membrane tethers. The tethers were individually anchored by multiple α_4_β_1_/VCAM-1 complexes that prolonged the extension of the viscous tethers. *In vivo*, the formation of these membrane tethers may provide the quantal structural units for the arrest of rolling monocytes within the blood vessels.

## Introduction

The recruitment of leukocytes from circulation involves a complex multistep adhesion cascade that is characterized by the initial cell attachment to the endothelium, rolling, arrest, firm adhesion and crawling, followed by transmigration. At the molecular level, these events are facilitated by an assortment of adhesion molecules, including selectins and integrins. Leukocyte adhesion is regulated by chemokines, cell-type specific signaling molecules expressed by the endothelium, that exert their effects through G-protein coupled receptor (GPCR) activation of integrins [1].

Circulating leukocytes are captured by the endothelium through the interactions of selectins and PSGL-1 [2]. Subsequent shear resistant leukocyte rolling involves the formation and breakage of tethered catch bonds [3]–[5] and “slings”, long membrane tethers with multiple discrete patches of PSGL-1, that wrap around the cell to form adhesive contacts in front of the rolling cell [6]. Under inflammatory conditions and in response to chemokine activation, rolling cells arrest in a process that involves both affinity and avidity modulation of the α_4_β_1_ integrin [7]–[11]. α_4_β_1_-mediated cell adhesion under flow also involves integrin clustering [1], the coupling of α_4_β_1_ to the actin cytoskeleton through paxillin [12], and actin polymerization via the activation of Rap1 and Rac [13].

Monocyte chemoattractant protein-1 (MCP-1, CCL2), an 8 kDa chemokine expressed on the surface of endothelial cells, recruits monocytes to the vascular endothelium (VE) in response to tissue damage signals and has been shown to contribute to the progression of monocyte related inflammatory diseases including atherosclerosis [14], diabetic nephropathy [15], allergic reactions [16] and neuronal inflammation in both peripheral [17] and central nervous systems [18]. Although it is well established that MCP-1 promotes cell adhesion, the biomechanical mechanism for cell arrest remains ill-defined. The current study investigates the mechanism by which immobilized MCP-1 promotes rapid integrin-mediated adhesion of THP-1, a monocytic cell line that expresses both α_4_β_1_ and LFA-1(Figure S1), as well as CCR2, the receptor for MCP-1 [19]–[21].

The atomic force microscope (AFM) was used to overcome two challenges in this investigation: the sub-second response of the leukocytes to chemokine [1] and the detection of weak intermolecular adhesive forces [22]. The AFM force measurements showed a significant increase in THP-1 adhesion to VCAM-1 co-immobilized with MCP-1 relative to VCAM-1 alone. Further analysis of the force measurements revealed that prolonged cell attachment induced by MCP-1 can be attributed to the formation of long membrane tethers, each supported by multiple high affinity α_4_β_1_/VCAM-1 complexes.

## Materials and Methods

### Cell Culture

THP-1 (ATCC #TIB-202) cells, a monocytic cell line that expresses of MCP-1 receptor levels comparable to human monocytes [16], [19]–[21], were maintained in RPMI-1640 (Atlanta Biologicals) supplemented with 10% fetal bovine serum and penicillin/streptomycin (50 U/mL/50 µg/mL CellGro) at 5% CO_2_ and 37°C. Cells were passaged every 48 hours and 12–24 hours before measurements.

### Immobilizing THP-1 cells onto the AFM cantilevers

Veeco MLCT-C cantilevers with a nominal spring constant 0.01 N/m were used in all measurements THP-1 cells were attached to AFM cantilevers coated with poly-L-lysine (PLL, Sigma P4832). Cantilevers were cleaned with acetone for 5 minutes and then UV irradiated for 10 minutes. Following UV irradiation, cantilevers were soaked briefly in 0.1 M NaHCO_3_ (pH 9.0) to ionize the surface of the cantilevers. The cantilevers were removed from the NaHCO_3_ solution, air dried, and immersed in 100 µl of PLL, (0.1 mg/mL) overnight at 4°C in a humidified chamber. After cantilevers were functionalized with PLL, they were rinsed three times in PBS and mounted on the AFM cantilever holder. Once the cantilever spring constant was determined [23], the cantilever was gently lowered on top of the cell until it just touched the cell long enough for the cell to adhere to the cantilever [24].

### Functionalizing substrates with purified proteins

Hydrophobic Petri dishes (Falcon # 35-1008) were used in all experiments. A 20 µL drop of NaHCO_3_ (pH 9.0) was briefly pipetted onto the center of each Petri dish. After removing NaHCO_3_, 20 µL of the human VCAM-1 Fc chimera (huICAM-1, R&D systems 720-IC-200) or human ICAM-1 Fc chimera (huVCAM-1, R&D systems 862-VC) was applied to the same spot and incubated overnight at 4°C in a humidified chamber. After coating overnight, the dish was rinsed three times in PBS, and blocked with 1% w/v Pluronic® F108NF Prill Poloxamer 338 (BASF) in PBS for 30 minutes.

### AFM measurements and analysis

All force measurements were conducted in cell culture medium and at 37°C using a laboratory built AFM equipped with a 90 µm closed loop piezoelectric translator (Physik Instrumente, P-841.60 & E-509 sensor module). THP-1 cells were attached to the tip of AFM cantilever as described above. For the whole cell measurements, Petri dishes were functionalized with huICAM-1 or huVCAM-1 at 0.5 µg/ml and huMCP-1 (Peprotech 300-04) at 5 µg/ml when needed. The scan speed of both approach and retraction in the whole cell force measurements was ∼3 µm/s at a sampling rate of ∼1,200 samples/sec and zero dwell time between consecutive measurements. However, since the cell-functionalized cantilever is retracted to a position ∼60 µm above the surface of the Petri dish to ensure breakage of the long membrane tethers, there is a 20 s delay between cell-substrate detachment and cell-substrate contact of consecutive measurements. At contact, the cell was pressed against the coated substrate with an indentation force of ∼500 pN for 100 ms.

**Figure 1:**
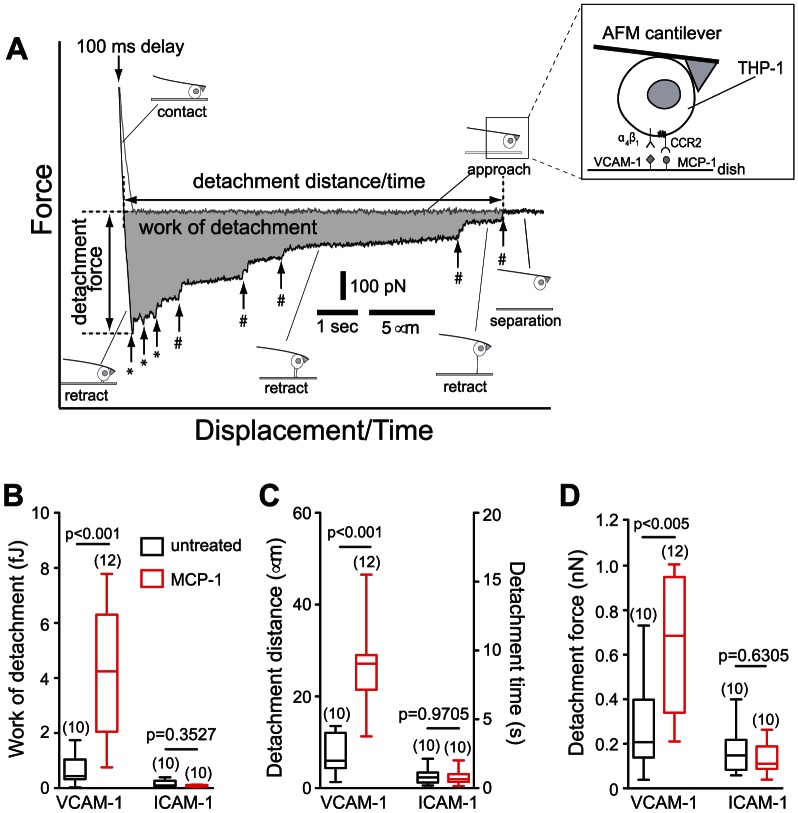
AFM measurement of THP-1 adhesion to immobilized adhesion molecules. (A) AFM force-displacement curve of THP-1 adhesion to VCAM-1 co-immobilized with MCP-1. The approach (gray) and retract (black) traces of the AFM measurement are shown. Shaded area represents the work of detachment done by the cantilever to completely detach the cell from the substrate. Vertical arrows denote rupture events mediated by cytoskeleton-anchored (*) or membrane-tethered integrins (#). The inset is a representation of the experimental system. A flexible AFM cantilever was used to immobilize a THP-1 cell expressing α_4_β_1_ and LFA-1 integrins and CCR-2 receptors of MCP-1, which was coimmobilized with ICAM-1 or VCAM-1 on the dish surface. Box-and-whisker plots of the (B) work of detachment, (C) detachment distance and detachment time, and (D) detachment force of THP-1 cell adhesion to VCAM-1 and ICAM-1, with and without co-immobilized MCP-1. The number in parenthesis above the plots corresponds to the number of cells studied. Numbers above each horizontal line represent the p-value from Mann-Whitney tests between respective measurement conditions. AFM measurements were acquired with a scan speed of ∼3 µm/s and a dwell time of 100 ms between approach and retract traces.

Rupture events in whole cell adhesion analysis were automatically determined from the local minima of the first derivative of the force curve using Igor Pro (Wavemetrics Inc., Lake Oswego, OR). Prior to taking the derivative, force curves were smoothed with a binomial smoothing window to remove high frequency noise. The work of detachment and total detachment distance were determined as shown in Figure 1A. The work of detachment was determined by integrating the force over the retraction distance. The total detachment distance and time represent the cantilever retraction distance required to completely detach the cell from the substrate and the time that the cell remains bound to the substrate, respectively. Jumps in the force curves preceded by a linear increase in force were considered as cytoskeleton anchored ruptures. Jumps preceded by a plateau with <15% derivation and persisting for >0.25 µm were considered as tethers [25].

For the single molecule force measurements, Petri dishes were functionalized with 0.5–2.5 µg/mL huICAM-1 or 0.25–1.0 µg/mL huVCAM-1 and 5 µg/mL huMCP1. To ensure that a high percentage of the measured adhesion reflected single molecule events, the adhesion frequency was maintained at <30% [26] by modulating the interaction time (0–0.3 s) and interaction force (50–200 pN). To vary the loading rate and tether extraction force of the single molecule measurements, measurements were conducted at retraction speeds of 2 to 25 µm/s and sampling rates of 800–10,000 samples/sec.

For single molecule data analysis, only force curves exhibiting a single rupture were considered as possible single molecule adhesion events. Single tethers were identified as adhesion events exhibiting a constant force plateau persisting for >0.25 µm [27]. Tether lifetime was computed by determining the time difference between the rupture point and the point in the force curve where 90% of the maximum tether extraction force was reached. Tether extraction force was taken as the force difference between the plateau and just after bond dissociation. Tether data were grouped by pulling speed and the cumulative tether lifetime probability was computed for each speed group. To determine the mean lifetime (*T*) of each speed group, cumulative tether lifetime probability was fit to a single exponential probability model 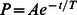 where *A* is a pre-exponential factor, and *t* is the single tether lifetime. Mean tether lifetimes for each speed group against the median tether force were fit to the Bell model: 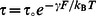, where 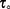 is the intrinsic bond lifetime, 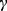 is the binding strength, *k*_B_ is Boltzmann's constant, and *T* = 310 K [28], [29].

Single molecule adhesion events not exhibiting a force plateau were considered force ramp rupture events. Single molecule rupture forces as function of loading rates were fitted using the Bell-Evans model: 
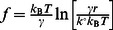
, where, 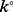 is the intrinsic dissociation rate (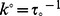) and *r* is the loading rate of the measurement [29].

All curve fitting was performed in IGOR Pro by minimizing chi-square statistic for the optimal fit. Unless otherwise stated, data are reported as mean ± standard error of the mean. Differences between the parameters calculated on untreated and MCP-1-stimulated cells and between control and blocked measurements were tested by two tailed Mann-Whitney test. Statistical significance was assumed at *p*<0.05. All statistical tests were performed in GraphPad Prism 5.0.

## Results

### MCP-1 promotes rapid adhesion of THP-1 cells to VCAM-1, but not to ICAM-1

To investigate the effects of MCP-1 on monocyte adhesion, we carried out direct measurements of cell-substrate interaction using the AFM. The inset of Figure 1A illustrates the design of our experimental system, which consists of a THP-1 cell attached to the end of a poly-L-lysine coated AFM cantilever and a Petri dish coated with VCAM-1 or ICAM-1. Interaction between the THP-1 cell and the immobilized protein was ascertained by monitoring the deflection of the AFM cantilever as described in Materials and Methods. To mimic the initial contact between monocytes and the vascular endothelium *in vivo*, adhesion measurements were conducted at 37°C and THP-1 cells were pressed against the functionalized substrate with an applied force of ∼500 pN for ∼100 ms [25]. Figure 1A presents an AFM force measurement carried out with a THP-1 cell coupled to the cantilever and a dish coated with both VCAM-1 and MCP-1. As shown in Figure 1B and Figures S2A&B, THP-1 adhered to immobilized VCAM-1 significantly stronger than to immobilized ICAM-1 as revealed by a >4-fold difference in the work of detachment. Moreover, co-immobilization of MCP-1 promoted adhesion of THP-1 to VCAM-1, but not to ICAM-1. The work required to detach MCP-1-stimulated THP-1 cells from immobilized VCAM-1 was 9 times higher than that of the untreated cells (Figure 1B), and did not involve changes in the expression of α_4_ or β_1_ in THP-1 cells (Figure S3). This near instantaneous activation of THP-1 adhesion by immobilized MCP-1 is consistent with previous report of subsecond tethering of leukocytes to VCAM-1 in response to the chemokine SDF-1 [1]. As evident by Figure 1C, the enhancement in cell adhesion can be attributed to the ability of stimulated cells to stretch and maintain contact with the substrate over distances of tens of microns.

THP-1 adhesion to immobilized VCAM-1 was inhibited by antibodies against β_1_, but not by function blocking antibodies against β_7_ (Figure S4A). Moreover, cell adhesion was suppressed by inhibition of phospholipase C (PLC), a component of the signaling pathway downstream of the GPCR (Figure S4B) [30], [31]. In addition, THP-1 cells did not adhere to Petri dishes coated with MCP-1 or Pluronic alone (Figure S2C). Together, these observations confirmed that MCP-1 promoted the specific adhesion of THP-1 cells to immobilized VCAM-1 via α_4_β_1_.

### THP-1 adhesion is mediated by cytoskeleton-anchored and membrane-tethered α_4_β_1_ integrins

Upon closer examination of the AFM cell adhesion measurements, it was evident that THP-1 cells detached from the ligand-functionalized surface via a series of ruptures. Each rupture, as revealed by the sharp vertical transitions of 20–50 pN, corresponded to the specific unbinding of one or more α_4_β_1_/VCAM-1 bonds. It is unlikely that these rupture events stemmed from the extraction of physisorbed VCAM-1 or α_4_β_1_ from the cell membrane since the forces associated with these processes are expected to be significantly greater than the rupture forces of an integrin-ligand complex [32]–[34]. The AFM measurements also revealed that THP-1 adhesion to immobilized VCAM-1 was mediated by two types of connections: (i) cytoskeleton -anchored and (ii) membrane-tethered linkages. The rupture of cytoskeleton-anchored α_4_β_1_/VCAM-1 complexes, which typically occurred within a few microns of cell-substrate separation, was preceded by the extension of elastic cytoskeletal elements and a linear increase in the pulling force. This interpretation of the AFM measurements is supported by prior studies showing that disruption of the actin cytoskeleton suppressed cytoskeleton-anchored bond formation [25], [35], [36]. By contrast, membrane-tethered α_4_β_1_/VCAM-1 ruptures exhibited a long force plateau prior to its dissociation. As shown in Figure S5, the tether extraction force increased linearly within the range of retraction speeds applied in the measurements [37]. This viscous force has been attributed mainly to the slip that occurs when the cell membrane flows over the cytoskeleton [35]–[40] and is not an intrinsic property of the α_4_β_1_/VCAM-1 complex.

**Figure 2:**
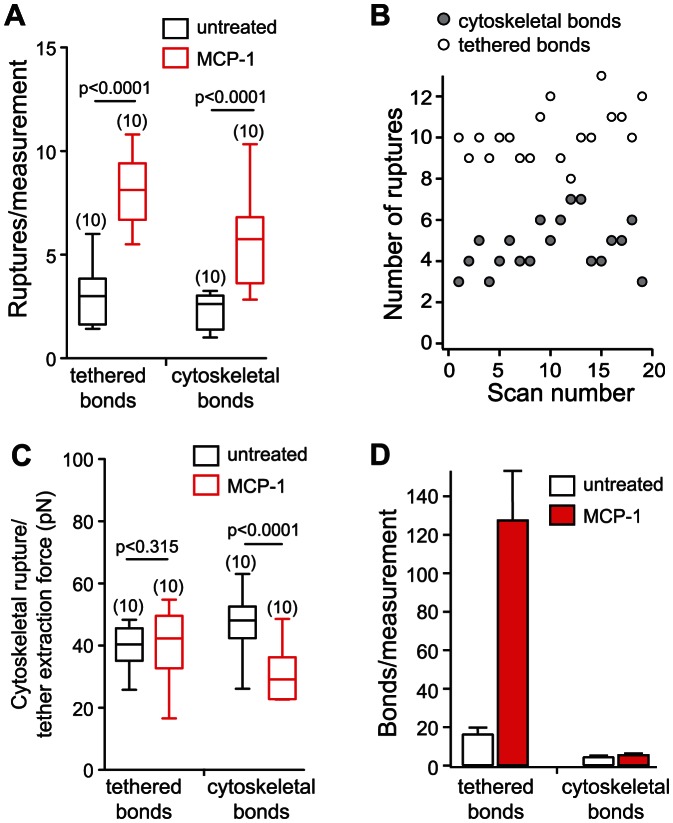
MCP-1 enhanced the number of cytoskeleton-anchored and membrane tethered α_4_β_1_/VCAM-1 ruptures per AFM measurement. (A) Box-and-whisker plot of the number of cytoskeleton-anchored and membrane-tether bond rupture events in AFM whole cell measurements with or without co-immobilized MCP-1. (B) Variation in the number of cytoskeleton-anchored and membrane-tethered ruptures from consecutive measurements. (C) Box-and-whisker plot of the extraction forces of membrane-tethers and cytoskeleton-anchored bonds rupture forces measured from whole cell adhesion measurements on untreated cells or cells stimulated with MCP-1.Measurements were acquired from 10 cells. Two-tailed Mann-Whitney tests were performed comparing untreated to stimulated cells. (D) Predicted number of cytoskeleton-anchored and membrane tethered α_4_β_1_/VCAM-1 bonds associated with the attachment of untreated and MCP-1stimulated cells to immobilized VCAM-1. Error bars assigned to membrane-tethered complexes were based on the estimated number of complexes per tether (Figure 5C). Error bars assigned to the cytoskeleton-anchored complexes were based on the s.e.m. of the number of cytoskeleton-anchored ruptures per measurements.

MCP-1 stimulation of THP-1 cells increased the occurrence of cytoskeleton-anchored and membrane-tethered ruptures by factors of 2.2 and 2.7, respectively (Figure 2A). Moreover, we did not observe any variation in the number of cytoskeleton-anchored and membrane-tethered ruptures in consecutive measurements on a same cell, suggesting that if integrin-cytoskeleton bonds were severed during the measurements, they reformed during the ∼20 s delay between measurements (Figure 2B). Interestingly, MCP-1 stimulation suppressed the forces associated with the rupture of cytoskeleton-anchored α_4_β_1_/VCAM-1 bonds, but not the tether extraction forces (Figure 2C and Figure S6). It should be noted that these measurements were carried out using VCAM-1 Fc chimera, a disulfide-linked homodimer, which may increase the probability for the formation dimeric α_4_β_1_/VCAM-1 complexes. The observation that the rupture force of the cytoskeleton-anchored α_4_β_1_/VCAM-1 bonds of untreated cells is approximately 2 times greater than the rupture forces of MCP-1 stimulated cells (Figure 2C) suggests that cytoskeleton-anchored α_4_β_1_ exists as dimers or larger aggregates in untreated THP-1 cells. When the cells are stimulated with MCP-1, the α_4_β_1_ aggregates dissociate into smaller independent units.

To determine if tether extraction force changes during cell detachment, we plotted the average extraction forces of the first, second and successive tethers to rupture in Figure S7. Untreated cells formed on average 3 tethers per measurement and seldom more than 5 tethers, while cells stimulated with MCP-1 formed greater number of tethers (∼8 tethers), up to 11 in some measurements. As shown, the extraction forces of the tethers of the untreated and MCP-1 stimulated cells did not change significantly from the first to last tether.

### Membrane tethers are supported by a high affinity form of α_4_β_1_

**Figure 3:**
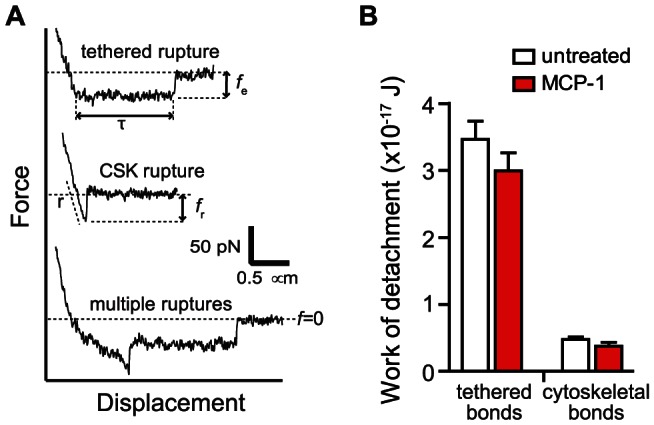
Single molecule measurements of the α_4_β_1_/VCAM-1 complex. (A) Representative force-distance curves were acquired during cantilever retraction (∼3 µm/s) following a contact time of 100 ms. (Top Trace) Single tethered rupture event. Quantification of tether lifetimes (*τ*) and extraction forces (*f*_e_) are depicted. (Middle Trace) Single cytoskeleton anchored rupture event. Loading rates (*r*) were determined from the linear slope of the force-distance curve immediately prior to the rupture, as indicated by the dashed line. Rupture force (*f*_r_) is determined from the peak of the rupture event to zero force (horizontal dash line). (Bottom Trace) Measurements exhibiting multiple ruptures. (B) Work of detachment (mean ± s.e.m.) for single molecule adhesion of a cytoskeleton-anchored and membrane-tethered α_4_β_1_/VCAM-1 complex.

To determine if changes in the energetics of the α_4_β_1_/VCAM-1 interaction contributed to the rapid increase in THP-1 adhesion following MCP-1 stimulation, single molecule force measurements were carried out to characterize the dynamic strength of individual α_4_β_1_/VCAM-1 complexes. Under the conditions (adhesion frequency <30%) that favored the detection of single molecule interaction, three types of adhesion events were observed: 1) single membrane-tethered bonds (Figure 3A, top trace), 2) single cytoskeleton-anchored bonds (middle trace), and 3) adhesion characterized by multiple ruptures (bottom trace). As summarized by Figure 3B, the work that was required to rupture a tethered complex was ∼10 fold greater than that of a cytoskeleton-anchored complex. Interestingly, the work of detachment of both tethered and cytoskeleton-anchored complexes was not significantly different between untreated and MCP-1 stimulated cells when only a single rupture was observed (Figure 3B).

**Figure 4:**
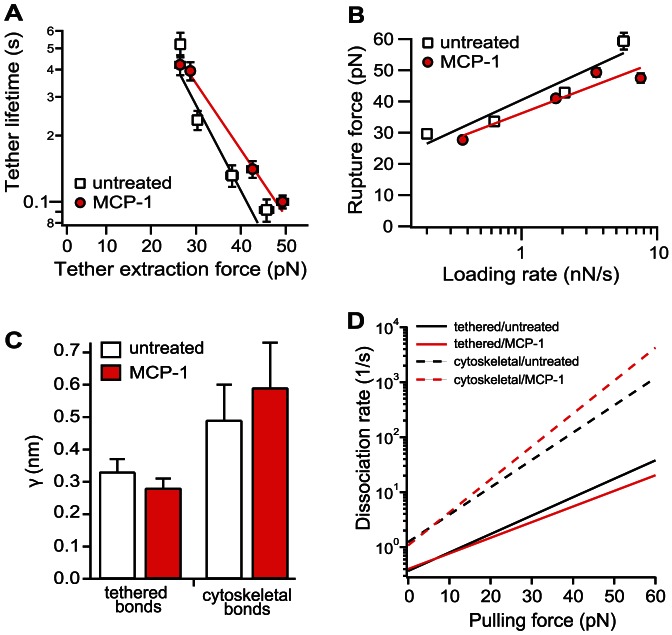
Effects of MCP-1 on single molecule membrane-tethered and cytoskeleton-anchored α_4_β_1_/VCAM-1 bonds. (A) Bond lifetime versus tether extraction force of untreated (squares) and MCP-1-stimulated (circles) α_4_β_1_/VCAM-1 single tethered ruptures. Force spectra of single tethered α_4_β_1_/VCAM-1 bonds were fitted with the Bell model. Error bars denote ± 1 standard error of the median tether force (horizontal) or ±1 s.e.m of the mean tether lifetimes (vertical). (B) Force spectra (rupture force versus loading rate) of untreated (squares) and MCP-1-stimulated (circles) single cytoskeleton α_4_β_1_/VCAM-1 anchored bonds. Data were fitted with the Bell-Evans model. (C) Plot of energy barrier width parameters for membrane-tethered and cytoskeleton-anchored α_4_β_1_/VCAM-1 complexes. (D) Kinetic profiles of untreated and MCP-1 stimulated cells. The force dependent dissociation rate of the complex is given by 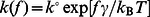, where *k*° and γ the Bell model parameters tabulated in Table 1. Single molecule measurements were conducted on a total of 27 cells for untreated condition, and a total of 24 MCP-1 treated cells. All of the data points in A and B were within their respective 95% confidence bands of the fits.

To characterize the dynamic properties of the individual tethered α_4_β_1_/VCAM-1 complex, its lifetime was determined as a function of tether extraction force by varying the pulling speed of the force measurement (Figure S8). The derived lifetime vs. pulling force relation (Figure 4A) revealed that the tethered α_4_β_1_/VCAM-1 complex is a slip bond within the range of pulling force generated in our measurements [28], though the α_4_β_1_/VCAM-1 complex may exhibit properties of a catch bond at lower forces [3], [41], [42]. To determine the intrinsic bond lifetime (τ_0_) and energy barrier width (γ) of the energy landscape of the tethered α_4_β_1_/VCAM-1 bond, the Bell model (i.e., 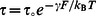) was fitted to the acquired data (Figure 4A) (Materials and Methods) [28]. *γ* estimates the resistance of the adhesive bond over a range of applied forces. A decrease in *γ* indicates that the adhesive interaction needs to overcome a steeper potential and is less responsive to a pulling force. MCP-1 stimulation of THP-1 cells did not result in a significant change in energy barrier width for tethered α_4_β_1_/VCAM-1 complexes (Figure 4C).

The dynamic strength of the cytoskeleton-anchored α_4_β_1_/VCAM-1 bond was determined by measuring the rupture force of the complex as a function of loading rate, *r* (Figures 4B & S9). Table 1 tabulated the derived lifetime and energy barrier width based on the best fit of the Bell-Evans model (i.e., 
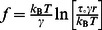
) to the acquired result (Materials and Methods) [29]. As with membrane-tethered bonds, MCP-1 stimulation had negligible effect on the strength of the cytoskeleton-anchored bond. A comparison of the current results to the values obtained by Zhang *et al.* [34] revealed that the cytoskeleton-anchored α_4_β_1_ of both untreated and MCP-1 stimulated cells was in the low affinity state (Table 1). Moreover, the model parameters showed that, when compared to cytoskeleton -anchored bonds, membrane-tethered bonds exhibited significantly shorter energy barrier widths (Figure 4C), which implies that the membrane-tethered bonds are more resistant to force (Figure 4D). Although the dissociation rates of the membrane-tethered bonds are slower (longer lifetimes) than that of cytoskeleton-anchored bonds, which also reflects higher affinity, this parameter is subjected to large uncertainty due to the extrapolation of the model to the rate axis. The energy barrier width parameter is less prone to uncertainty and it is clearly smaller for tethered bonds. Thus, we can conclude that membrane-tethered bonds exist in a higher affinity state and can better resist a pulling force than cytoskeleton-anchored bonds (Figure 4D).

**Table 1:** Bell model parameters of the membrane-tethered and cytoskeleton-anchored *α*_4_ *β*_1_/VCAM-1 complexes.

Ligand-receptor pair	Conditions	*γ* (Å)	*k*° (s^−1^)
Membrane-tethered *α*_4_ *β*_1_/VCAM-1	Untreated	3.3±0.4	0.37±0.12
	MCP-1	2.8±0.3	0.40±0.08
cytoskeleton-anchored *α*_4_ *β*_1_/VCAM-1	Untreated	4.9±1.1	1.24±0.45
	MCP-1	5.9±1.4	1.08±0.90
cytoskeleton-anchored *α*_4_ *β*_1_/VCAM-1*	Resting	5.2	1.1
	TS2/16-activated	6.2	0.04
*Purified α*_4_ *β*_1_-Fc/VCAM-1*	Mg^2+^-activated	5.9	0.13

The Bell model parameters were determined as described in Methods.

* Values were obtained from Zhang *et al.* [34].

### Membrane tethers are supported by multiple α_4_β_1_/VCAM-1 complexes

**Figure 5:**
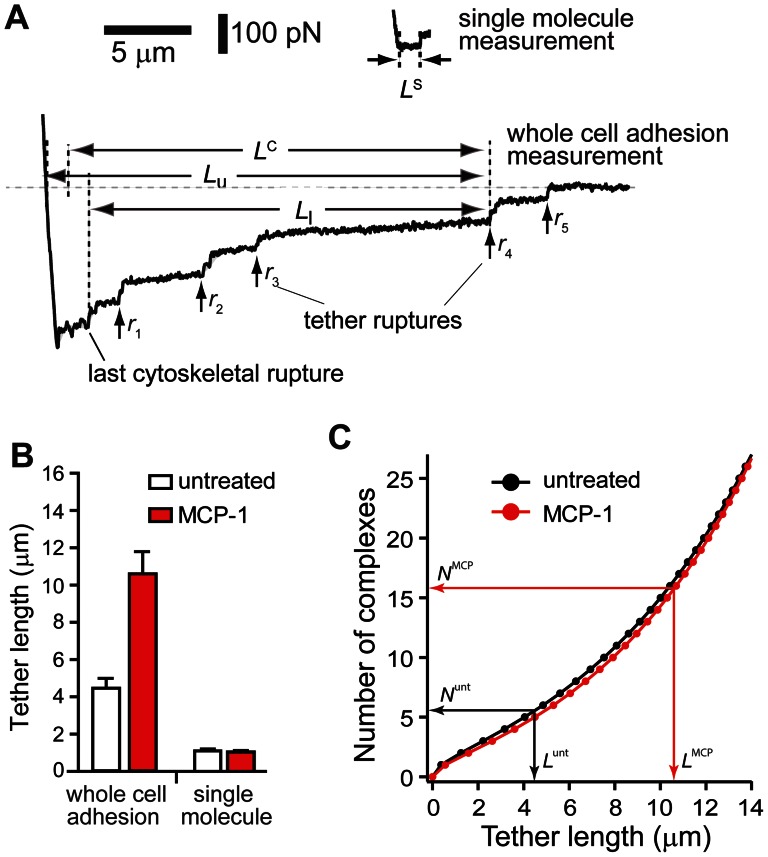
Individual tethers in whole cell measurements were supported by multiple α_4_β_1_/VCAM-1 complexes. (A) Comparison of AFM force measurements acquired with extensive (whole cell measurement) and minimal (single molecule measurement) cell-substrate contact. *L* ^S^ is the length of tethers supported by a single α_4_β_1_/VCAM-1 complex as detected in single molecule measurements. In whole cell adhesion measurement shown, the cell detachment process involved the formation and breakage of at least 5 membrane tethers, labeled *r*_1_ through *r*_5_. Each tether is suppoted by multiple α_4_β_1_/VCAM-1 complexes. The lower and upper limits (*L*_l_ and *L*_u_), and the average length (*L* ^C^) of fourth tether (*r*_4_) are shown. (B) Comparison of average tether lengths from whole cell measurements (*L* ^C^) and tether lengths from single molecule measurements (*L* ^S^). (C) Estimates of the number of α_4_β_1_/VCAM-1 complexes in a tether generated during the whole cell measurements. Black and red plots provide the range for the number of α_4_β_1_/VCAM-1 complexes in a single tether for untreated and MCP-1 stimulated cells, respectively. The superscripts *unt* and *MCP* associated with *N* (number of complexes) and *L* (tether length) designate untreated and MCP-1-stimulated cells, respectively.

The tethers detected in the whole cell adhesion measurements were noticeably longer than those of the single molecule measurements acquired at comparable retraction speed of ∼3 µm/s (Figure 5A). The average length of the tethers (*L^C^* in Figure 5A) generated in the cell adhesion experiments was on average 4.5 µm for untreated cells and 10.6 µm for MCP-1 stimulated cells (Figure 5B). These values were derived from average of the lower limit (*L*_l_) and upper limit (*L*_u_) of the tethers as shown in Figure 5A. *L*_l_ is the distance between the position of the tether breakage and the last cytoskeletal-anchored bond to break or the start of the plateau region if the first rupture happens to be a tether. *L*_u_ is the distance between the position of the tether breakage and the position of cell-substrate contact. By comparison, the tether length of both untreated and MCP-1 stimulated cells acquired from single molecule measurements was ∼1 µm. An explanation for this discrepancy is that there were multiple α_4_β_1_/VCAM-1 complexes supporting the individual tethers in the whole cell measurements. For bonds acting in parallel in a tether, lifetime (*T*) and length (*L*) of tether as a function of the number of α_4_β_1_/VCAM-1 complexes *N* are given by 
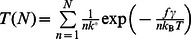
 and 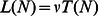, respectively, where *f* is the viscous force of the tether and *v* is retraction speed [43]. For the whole cell force measurements, *v* is 3 µm/s and *f* is ∼40 pN for both untreated and MCP-1 stimulated THP-1 cells (Figure 2C). Values for the Bell model parameters, 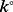 and 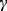, are given in Table 1. Hence, based on the average tether length, we estimate that the number of α_4_β_1_/VCAM-1 complexes anchoring individual tethers in untreated and MCP-1-stimulated THP-1 cells to be 5.5 and 15.8 complexes, respectively (Figure 5C).

## Discussion

The current study investigated early events in monocyte arrest following chemokine activation. Under conditions that mimicked the brief contact of circulating monocytes with the endothelium surface, with integrin ligands and chemokine co-immobilized on the same substrate, it was revealed that α_4_β_1_ is the predominant integrin responsible for the rapid adhesion of both untreated and MCP-1 stimulated THP-1 cells (Figure 1B). This result is consistent with previous observations that α_4_ integrins are localized with GPCRs in the microvilli tips, while β_2_ integrin subunits were found in the invaginated membrane [44] and therefore less accessible in the timescale (100 ms) and minimal indentation force of our AFM measurements.

Also consistent with the localization of α_4_β_1_ to the microvilli is the observation that a majority of α_4_β_1_ integrins involved in initiating adhesion are associated with long tethers formed by the cell membrane. Figure 2D plotted our estimates of the number of membrane-tethered and cytoskeleton-anchored α_4_β_1_/VCAM-1 bonds associated with the attachment of THP-1 cells to immobilized VCAM-1 in the whole cell measurements. These values were derived from the product of the number of membrane-tethered (or cytoskeleton-anchored) ruptures per measurement and the number of α_4_β_1_/VCAM-1 complexes associated with each rupture event. Based on the estimate that there were on average 5.5 and 15.8 bonds supporting each tether for the untreated and MCP-1 stimulated cells, respectively (Figure 5C), we estimated that there were a total of 16.5 and 126 membrane-tethered bonds supporting cell attachment during whole cell measurements for the untreated and MCP-1 stimulated cells, respectively (Figure 2D). To estimate the number of cytoskeleton-anchored bonds involved in the whole cell measurement, the cytoskeleton-anchored rupture force values acquired in the whole cell measurements (Figure 2C) were compared to the single molecule unbinding force of the α_4_β_1_/VCAM-1 complex (Figure 4B) acquired under comparable conditions. For MCP-1 stimulated cells, the cytoskeleton-anchored rupture force (∼28 pN) is less than the unbinding force of the unitary complex (∼30 pN) and, therefore, it is likely that each cytoskeleton-anchored rupture in the whole cell measurements involved the unbinding of a single α_4_β_1_/VCAM-1 complex. For the untreated cells, the cytoskeleton-anchored rupture force (∼48 pN) was greater than the single molecule unbinding force, but by less than a factor of 2 (Figures 2C and 4B). We estimate that each cytoskeleton-anchored rupture involved perhaps 2 complexes. Using these estimates for the number of cytoskeleton-anchored bonds per rupture, we estimate that there were ∼5–6 cytoskeleton-anchored complexes per measurement for both untreated and stimulated cells and thus significantly less than the number of membrane-tethered complexes (Figure 2D).

Figure 2D also showed that MCP-1 stimulation of THP-1 cells resulted in a 6-fold increase in the total number of α_4_β_1_/VCAM-1 bonds formed during the AFM measurements. Since the contact duration (100 ms) of the measurements was significantly shorter than the measured off-rates of the α_4_β_1_/VCAM-1 complexes (0.8–4 s), the increase in the number of α_4_β_1_/VCAM-1 complexes detected with MCP-1 stimulated cells can be attributed to an increase in the effective on-rate for the formation of the complex. Potential mechanisms of MCP-1 facilitated increase in the effective on-rate of α_4_β_1_/VCAM-1 complex formation include an upregulation of α_4_β_1_ affinity, an increase in cell surface expression, and changes in cellular mechanics [9], [25], [45]–[47]. Based on the single molecule measurements, it is unlikely that the increase in α_4_β_1_/VCAM-1 complexes formed was due to a simple upregulation in integrin affinity since the derived off-rates of the α_4_β_1_/VCAM-1 complex were essentially the same for both untreated and MCP-1 stimulated cells. However, it should be noted that the AFM measurements might not detect the conversion of a bent to an extended conformation of α_4_β_1_ [7] if the bent conformation is inaccessible to immobilized VCAM-1. As for upregulation in integrin expression, our flow cytometry studies showed no change in the surface expression of α_4_β_1_ between untreated and MCP-1 stimulated cells (Figure S3). Moreover, it is unlikely that the difference in the number of α_4_β_1_/VCAM-1 complexes is due to changes in in cellular mechanics since there was no statistical difference in Young's modulus (∼100 Pa.) of untreated and stimulated cells (Figure S10).

While the number and distribution of cytoskeleton-anchored and membrane-tethered α_4_β_1_/VCAM-1 complexes formed are dependent on the activation state of the THP-1 cell, the AFM measurements revealed a strong correlation between the affinity state of the α_4_β_1_/VCAM-1 complex and its mechanical linkage to THP-1 cells that is independent of MCP-1 stimulation (Figure 4D). Specifically, lower affinity α_4_β_1_ were anchored to the cytoskeleton, while a higher affinity form of α_4_β_1_ was anchored to the plasma membrane. This suggests that MCP-1 stimulation may decouple the cortical cytoskeleton from the plasma membrane, which then releases α_4_β_1_ from cytoskeletal constraints to express a high affinity conformer. Alternatively, MCP-1 stimulation may result in the formation of α_4_β_1_/VCAM-1 complexes that were stronger than the intracellular bonds that attached the cell membrane to the cytoskeleton. Although neither possibility can be definitively ruled out, it is likely that PLC plays a critical role in both models. PLC activation in response to chemokine stimulation has been shown to upregulate the affinity expression of α_4_β_1_ via a signaling pathway that also involves inositol 1,4,5-triphosphate receptors and increased intracellular calcium [30]. Moreover, it was shown that chemokine stimulation can induce the release of lymphocyte membrane from the cortical cytoskeleton via the inactivation of ezrin/radixin/moesin (ERM) proteins [48], [49]. The mechanism for release of ERM proteins from the plasma membrane involved the activation of PLC and the hydrolysis of phosphatidylinositol 4, 5-bisphosphate (PIP2). Consistent with these studies, our force measurements revealed that tether formation in response to MCP-1 stimulation was suppressed by PLC inhibition (Figure S4B).

The AFM measurements provided a “snapshot” of changes in cell mechanics within 100 ms of chemokine activation. As reported by Grabovsky *et al.*, this provided sufficient time for immobilized chemokine to induce integrin clustering and tether formation [1]. Consistent with the earlier report, our AFM measurements revealed that monocyte arrest following MCP-1 activation can be attributed to a decoupling of α_4_β_1_ from the cytoskeleton that promotes the formation of long membrane tethers. Unlike a braking mechanism involving elastic elements, where anchored bonds are rapidly disrupted by an increasing load, membrane tethers supported by multiple α_4_β_1_/VCAM-1 complexes allow for a sustained and continuous dissipation of energy and prolonged durations of adhesive contact. Since an applied force is distributed evenly among the viscous tethers, they are able to support a load for longer duration than the bonds independently anchored to the cytoskeleton (Figure S11). In addition, a reduction in membrane tether viscosity with tether extension further favors prolonging the bond lifetimes of the α_4_β_1_/VCAM-1 complexes and, thus, the formation of longer tethers. *In vivo*, this self-regulatory biomechanical mechanism allows the tethered cells to remain attached to the endothelium for longer duration to stretch and survey the endothelial microenvironment for additional signals and adhesion molecules. Remarkably, this process takes place in a very short time scale from 100 ms to only few seconds.

## Supporting Information

Figure S1 **Expression patterns of integrins, α_4_β_1_ and LFA-1 (α_L_β_2_) on THP-1 cells.** (A) Orange and green curves indicate FACS histogram plots of cells stained directly with FITC-labeled mAb against α_4_ (Ancell Corporation, Cat. # 200-040) and α_L_ (Ancell Corporation, Cat. # 158-040), respectively. Gray curve represents FACS histogram plots of cells stained with an isotype-matched antibody (Ancell Corporation, Cat. # 278-040). (B) Maximum projection images of THP-1 cells labeled with FITC-anti- α_4_ (left) and FITC-anti- α_L_ (right). The bar is 5 µm. Confocal images were acquired on a Nikon A1R microscope in 16 sections at 0.75 µm intervals.
(TIF)

Figure S2 **AFM force-displacement measurements of THP-1 adhesion to immobilized ligands.** (A) Representative AFM force-displacement curves of THP-1 adhesion to immobilized VCAM-1 alone (left) and to VCAM-1 co-immobilized with MCP-1 (right). (B) AFM force-displacement curves of THP-1 adhesion to immobilized ICAM-1 alone (left) and to ICAM-1 co-immobilized with MCP-1 (right). (C) AFM force-displacement curves of THP-1 adhesion to immobilized MCP-1 alone (left) and to a Petri dish treated with Pluronic (right). The approach (gray) and retract (black) traces of the AFM measurement are shown.
(EPS)

Figure S3 **Flow cytometric analysis of THP-1 cell surface expression of integrin subunits binding to VCAM-1.** THP-1 cells stained for (A) α_4_ (B) β_1_ (C) β_7_ integrin subunits. Isotype control (negative), untreated (green), MCP-1 stimulated THP-1 cells (red). Cells were either untreated or treated with soluble MCP-1 (sMCP-1) for 10 minutes at 37°C. Fc receptors were blocked with Fcγ receptor binding inhibitor (eBioscience 14-9161-71). Cells were stained in flow cytometry staining buffer (eBioscience 00-4222-57) with the following integrin subunit antibodies: PE-α_4_ (eBioscience, clone 9F10, mouse IgG1, κ), PerCP-β_1_ (eBioscience, clone TS2/16, mouse IgG1, κ), FITC-β_7_ (BioLegend, clone FIB504, rat IgG2a, κ) and isotype controls, PE-mouse IgG1, κ (eBioscience, 12-4714-71) and FITC-rat IgG2a, κ (eBioscience, 11-4321-71). Cells were fixed with 2% paraformaldehyde. Flow cytometry was performed on a FACS Calibur flow cytometer (BD Biosciences), and histograms of plotting counts versus mean channel fluorescence were constructed using CellQuest software (BD Biosciences). The integrins, α_4_β_1_ and α_4_β_7_ are both receptors for VCAM-1. The flow cytometric analysis revealed that THP-1 cells expressed α_4_ and β_1_, but not β_7_.
(TIF)

Figure S4 **THP-1 adhesion to VCAM-1 co-immobilized with MCP-1 was suppressed by anti-β_1_ antibody and U-73122, an inhibitor of phospholipase C (PLC).** (A) THP-1 adhesion was inhibited by anti-β_1_ (R&D Systems, MAB17781) at 10 µg/ml, but not by 25 µg/mL anti-β_7_ (Biolegend, 321218) antibodies. (B) THP-1 adhesion was inhibited by U73122 (10 µM, Sigma-Aldrich), but not by U73343 (10 µM, Sigma-Aldrich), an inactive form of U73122 [31]. The work of detachment was quantified from whole cell adhesion measurements as described in the text. A series of reference measurements were initially acquired in the absence of the test agents. Cells were then treated with the mAbs or PLC inhibitors for 10 minutes, and a second series of adhesion measurements were acquired. Relative work of detachment is normalized to measurements acquired in the absence of the test agents.
(TIF)

Figure S5
**Viscous properties of membrane tethers supported by a single α_4_β_1_/VCAM-1 complex.** Median tether extraction forces plotted against the average pulling speed. Untreated (squares) and MCP-1 (circles) stimulated single α_4_β_1_/VCAM-1 tethered bonds. Vertical error bars denote ± standard error of the median. As shown, within the applied range of rates, tether force can be considered to increase linearly with retraction speed [37]. Thus, we used a phenomenological model (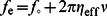) for the viscous extension of the lipid tethers. This model relates the extraction force on the tether to the retraction speed (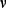) and can be used to estimate the effective viscosity (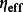) of individual tethers (0.15 pN.s/µm), and the threshold force ( *f*_o_∼25 pN) required to extract the tether. MCP-1 had no measurable effect on the properties of individual tethers.(TIF)

Figure S6 **Force histograms of the extraction forces of membrane-tethers (A) and rupture forces of cytoskeleton-anchored bonds (B) measured from whole cell adhesion measurements on untreated cells or cells stimulated with MCP-1.**
(TIF)

Figure S7 **Quantification of individual tether extraction forces in whole cell adhesion curves.** Each point represents the average tether extraction force for each observed tether in the whole cell adhesion curve. The tether number corresponds to the chronological occurrence of the tether. Error bars are s.e.m. A total of 61 force measurements from 10 cells were used to generate the untreated cell plot. 41 force measurements from 10 cells were used to generate the MCP-1-stimulated cell plot.
(TIF)

Figure S8 **Lifetimes of a tether anchored α_4_β_1_/VCAM-1 complex measured at pulling speeds of 2, 5, 15, 25 µm/s of untreated (A) and MCP-1-stimulated (B) THP-1 cells.** Dashed lines represent the upper and lower bounds of the 95% confidence intervals of the best fit to the cumulative lifetime probability (see Materials and Methods in the main text for more details).
(EPS)

Figure S9 **Single molecule α_4_β_1_/VCAM-1 force histograms of THP-1 adhesion to immobilized VCAM-1 either untreated (unfilled) or exposed to MCP-1 (red).** The loading rates (*r*) of the force measurements are given with the histograms.
(EPS)

Figure S10
**Young's modulus of untreated and MCP-1 stimulated THP-1 cells.** Young's modulus was determined by fitting the Hertz model of an elastic sphere pressed against a flat surface to the approach trace of the AFM force-indentation (*F*-δ) curves; 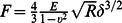, where *R* is the radius of the cell, *E*, Young's modulus and 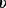, Poisson's ratio, assumed to be 0.5 (incompressible sample) [50]. The radius of THP-1 was determined by confocal microscopy to be ∼5 µm.(EPS)

Figure S11
**Biomechanical model for movement and arrest of monocytes.** (A) A spherical cell attached by a long tether to a flat substrate is subjected to a drag force, *F*_S_, that is proportional to the shear rate G: 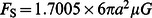, where *a* is the cell radius and *μ*, the medium viscosity [5], [51], [52]. The lateral velocity of the cell is governed by the balance between shear force and the horizontal component of the force required to extract *N* tethers: 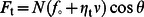, where *f*_0_ and *η*_t_ are the threshold extraction force and viscosity of the tether, respectively (see Figure S6) and *θ* is the angle formed by the tethers and the substrate. (B) The lateral velocity of the tethered cell depends on the number of tethers and is given by 
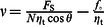
. For the plot presented, 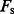 = 150 pN, *θ* = π/4, 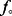 = 25 pN, and 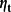 = 1 pN-s/µm. Activation by MCP-1 increases the number of tethered linkages, which reduces the velocity of the cell. More tethers will also reduce the load on the individual tethers and prolong the lifetime of the tethers. Chemokine activation also increases the number the integrin-ligand complexes supporting individual tethers, which further prolong the tether survival time. The reduced velocity and extended survival time of tethers enable the cell to remain in contact with the endothelium and more tethered linkages to form until the cell is arrested.(EPS)
