# Supplementary figures and images for: Elongated Membrane Tethers, Individually Anchored by High Affinity α4β1/VCAM-1 Complexes, Are the Quantal Units of Monocyte Arrests
Source: PLoS One. 2013 May 17;8(5):e64187. doi: 10.1371/journal.pone.0064187 (PMC3656870; doi:10.1371/journal.pone.0064187)

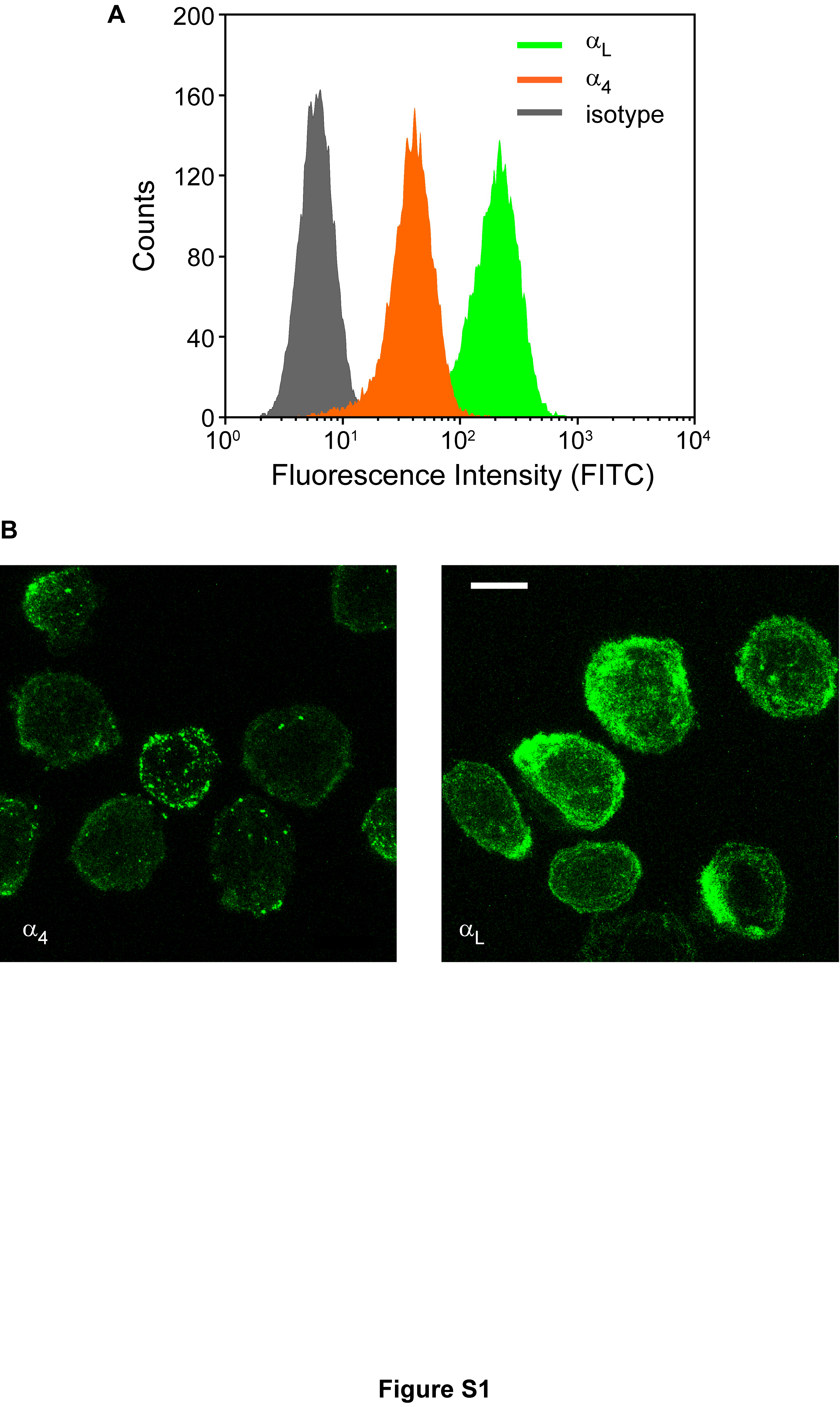

Supplement: Figure S1 — Expression patterns of integrins, α4β1 and LFA-1 (αLβ2) on THP-1 cells. (A) Orange and green curves indicate FACS histogram plots of cells stained directly with FITC-labeled mAb against α4 (Ancell Corporation, Cat. # 200-040) and αL (Ancell Corporation, Cat. # 158-040), respectively. Gray curve represents FACS histogram plots of cells stained with an isotype-matched antibody (Ancell Corporation, Cat. # 278-040). (B) Maximum projection images of THP-1 cells labeled with FITC-anti- α4 (left) and FITC-anti- αL (right). The bar is 5 µm. Confocal images were acquired on a Nikon A1R microscope in 16 sections at 0.75 µm intervals. (TIF) [file pone.0064187.s001.tif]

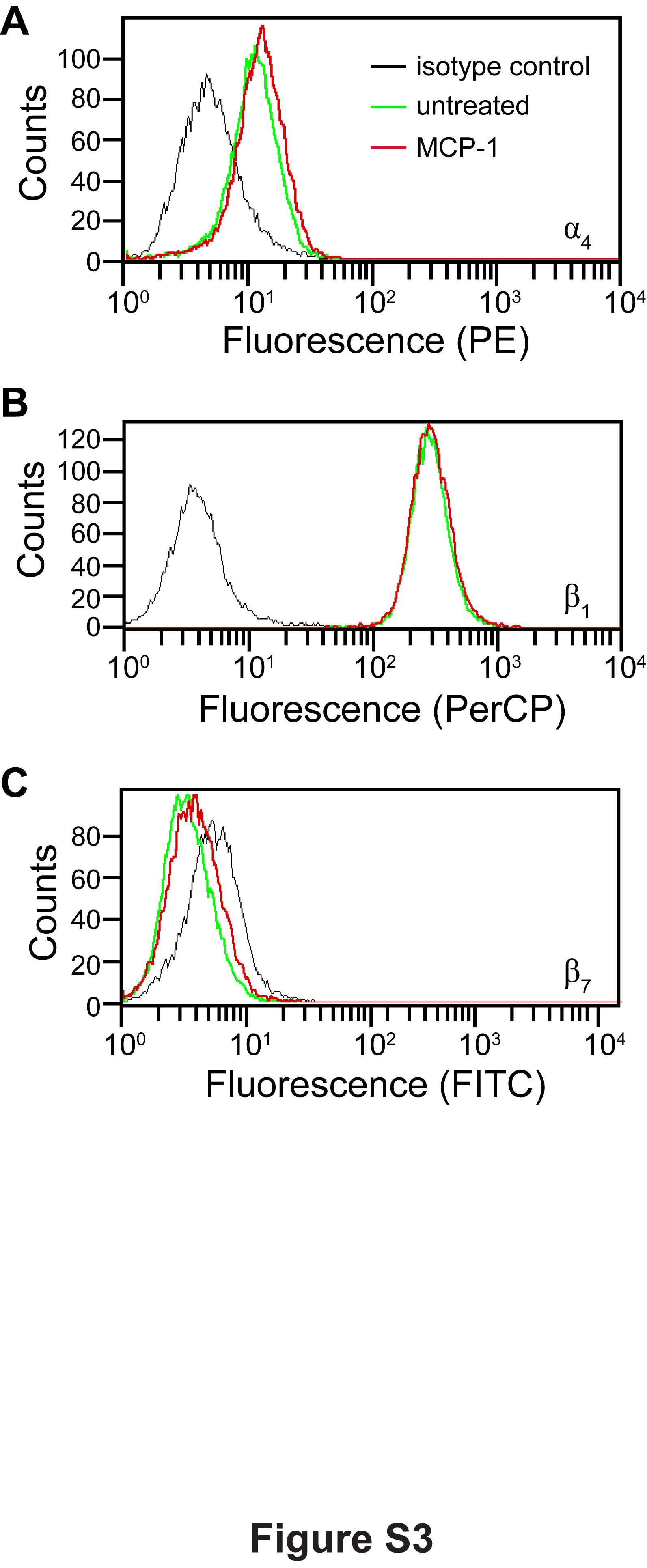

Supplement: Figure S3 — Flow cytometric analysis of THP-1 cell surface expression of integrin subunits binding to VCAM-1. THP-1 cells stained for (A) α4 (B) β1 (C) β7 integrin subunits. Isotype control (negative), untreated (green), MCP-1 stimulated THP-1 cells (red). Cells were either untreated or treated with soluble MCP-1 (sMCP-1) for 10 minutes at 37°C. Fc receptors were blocked with Fcγ receptor binding inhibitor (eBioscience 14-9161-71). Cells were stained in flow cytometry staining buffer (eBioscience 00-4222-57) with the following integrin subunit antibodies: PE-α4 (eBioscience, clone 9F10, mouse IgG1, κ), PerCP-β1 (eBioscience, clone TS2/16, mouse IgG1, κ), FITC-β7 (BioLegend, clone FIB504, rat IgG2a, κ) and isotype controls, PE-mouse IgG1, κ (eBioscience, 12-4714-71) and FITC-rat IgG2a, κ (eBioscience, 11-4321-71). Cells were fixed with 2% paraformaldehyde. Flow cytometry was performed on a FACS Calibur flow cytometer (BD Biosciences), and histograms of plotting counts versus mean channel fluorescence were constructed using CellQuest software (BD Biosciences). The integrins, α4β1 and α4β7 are both receptors for VCAM-1. The flow cytometric analysis revealed that THP-1 cells expressed α4 and β1, but not β7. (TIF) [file pone.0064187.s003.tif]

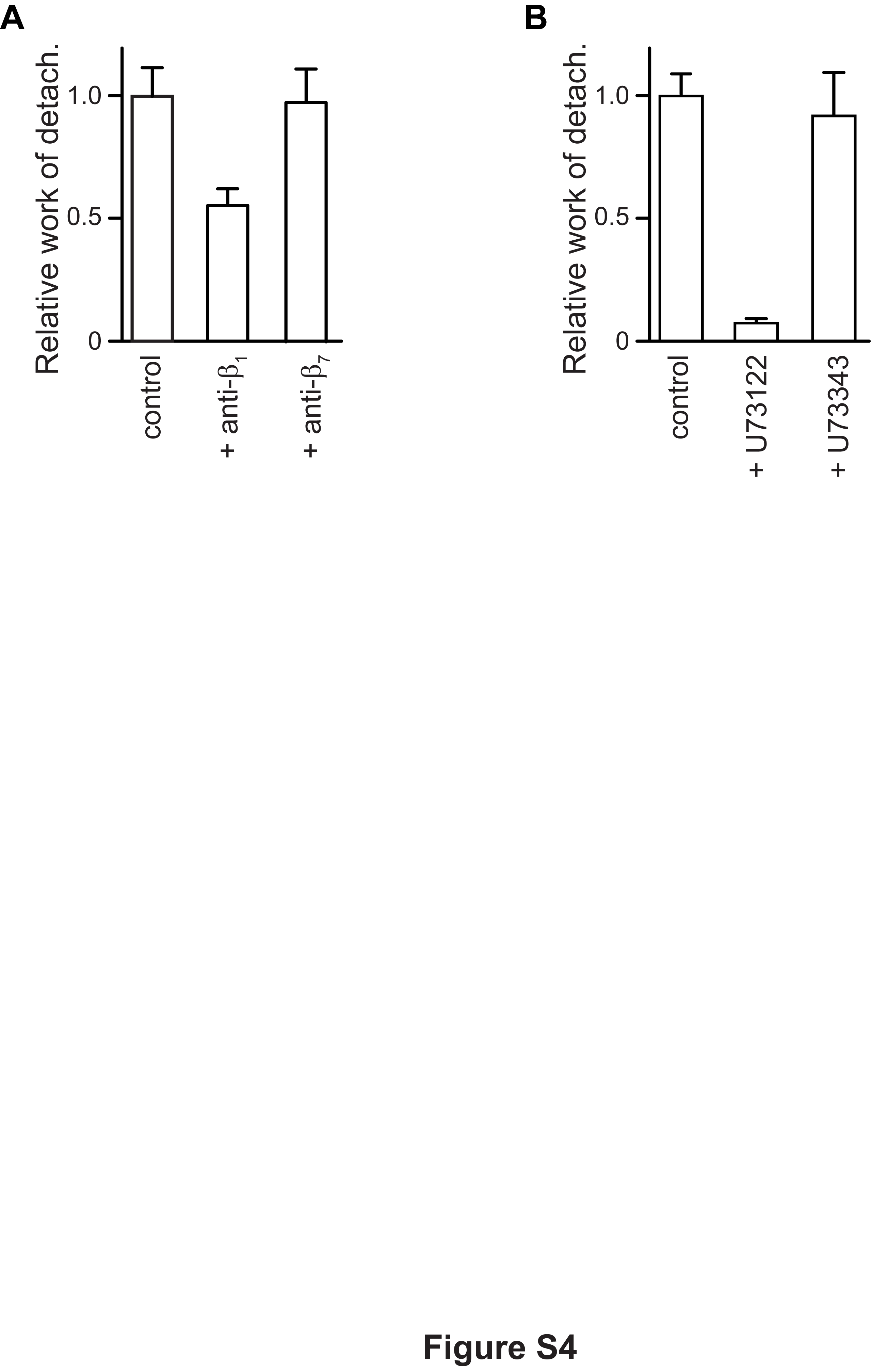

Supplement: Figure S4 — THP-1 adhesion to VCAM-1 co-immobilized with MCP-1 was suppressed by anti-β1 antibody and U-73122, an inhibitor of phospholipase C (PLC). (A) THP-1 adhesion was inhibited by anti-β1 (R&D Systems, MAB17781) at 10 µg/ml, but not by 25 µg/mL anti-β7 (Biolegend, 321218) antibodies. (B) THP-1 adhesion was inhibited by U73122 (10 µM, Sigma-Aldrich), but not by U73343 (10 µM, Sigma-Aldrich), an inactive form of U73122 [31]. The work of detachment was quantified from whole cell adhesion measurements as described in the text. A series of reference measurements were initially acquired in the absence of the test agents. Cells were then treated with the mAbs or PLC inhibitors for 10 minutes, and a second series of adhesion measurements were acquired. Relative work of detachment is normalized to measurements acquired in the absence of the test agents. (TIF) [file pone.0064187.s004.tif]

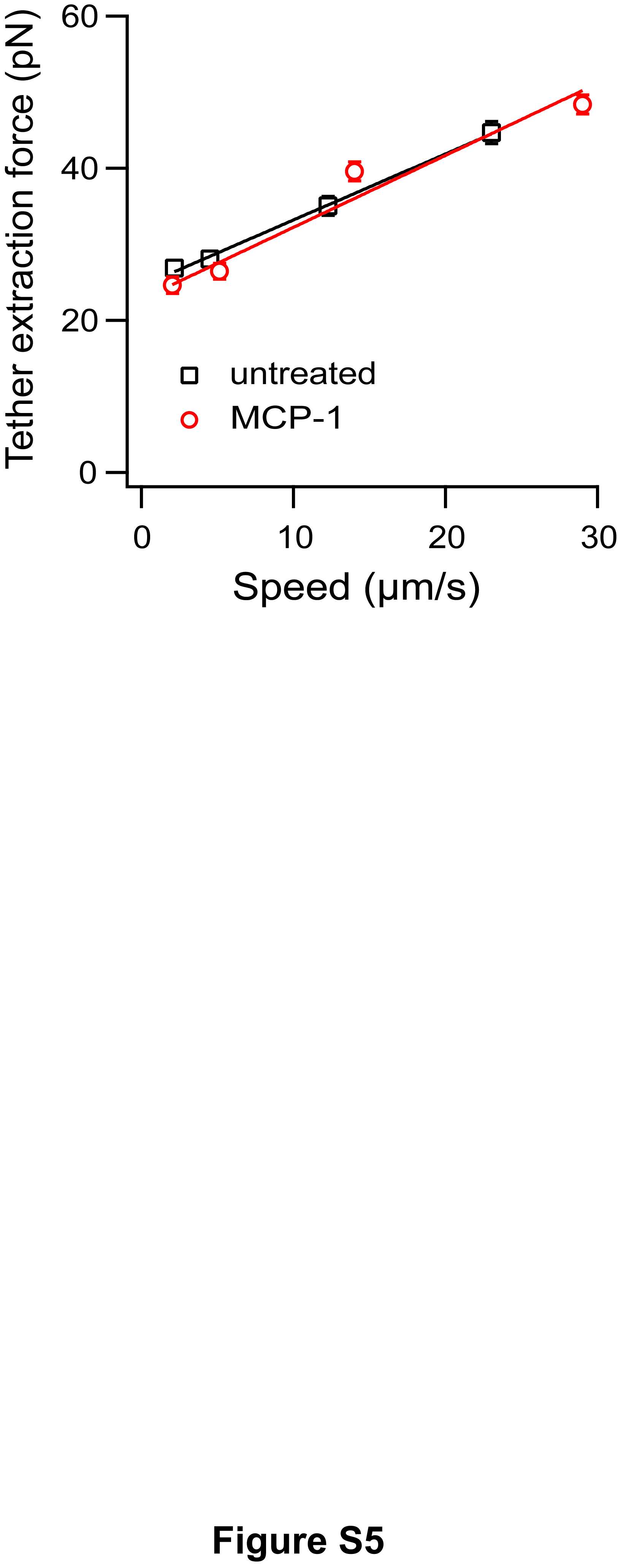

Supplement: Figure S5 — Viscous properties of membrane tethers supported by a single α4β1/VCAM-1 complex. Median tether extraction forces plotted against the average pulling speed. Untreated (squares) and MCP-1 (circles) stimulated single α4β1/VCAM-1 tethered bonds. Vertical error bars denote ± standard error of the median. As shown, within the applied range of rates, tether force can be considered to increase linearly with retraction speed [37]. Thus, we used a phenomenological model () for the viscous extension of the lipid tethers. This model relates the extraction force on the tether to the retraction speed () and can be used to estimate the effective viscosity () of individual tethers (0.15 pN.s/µm), and the threshold force ( f o∼25 pN) required to extract the tether. MCP-1 had no measurable effect on the properties of individual tethers. (TIF) [file pone.0064187.s005.tif]

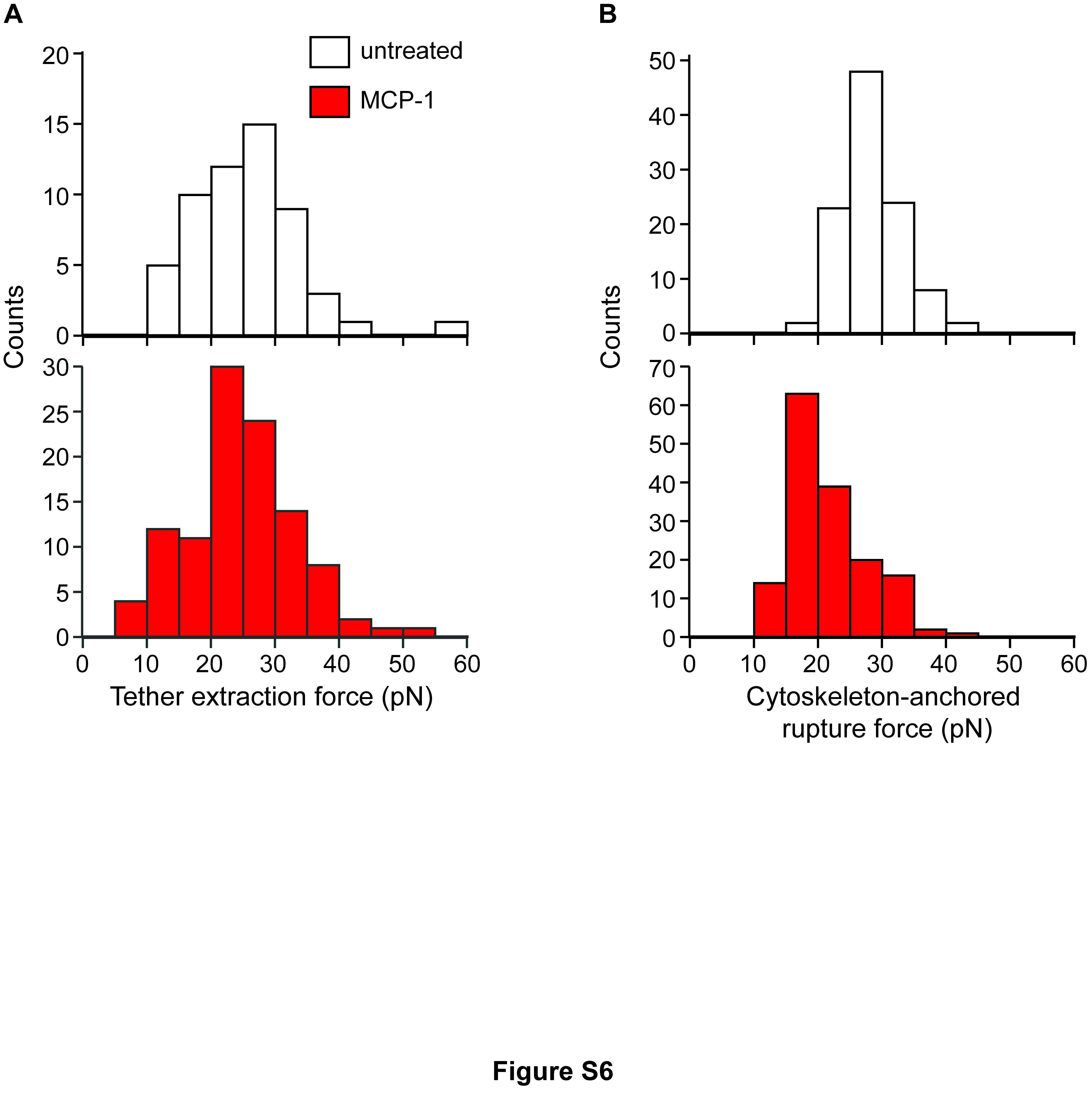

Supplement: Figure S6 — Force histograms of the extraction forces of membrane-tethers (A) and rupture forces of cytoskeleton-anchored bonds (B) measured from whole cell adhesion measurements on untreated cells or cells stimulated with MCP-1. (TIF) [file pone.0064187.s006.tif]

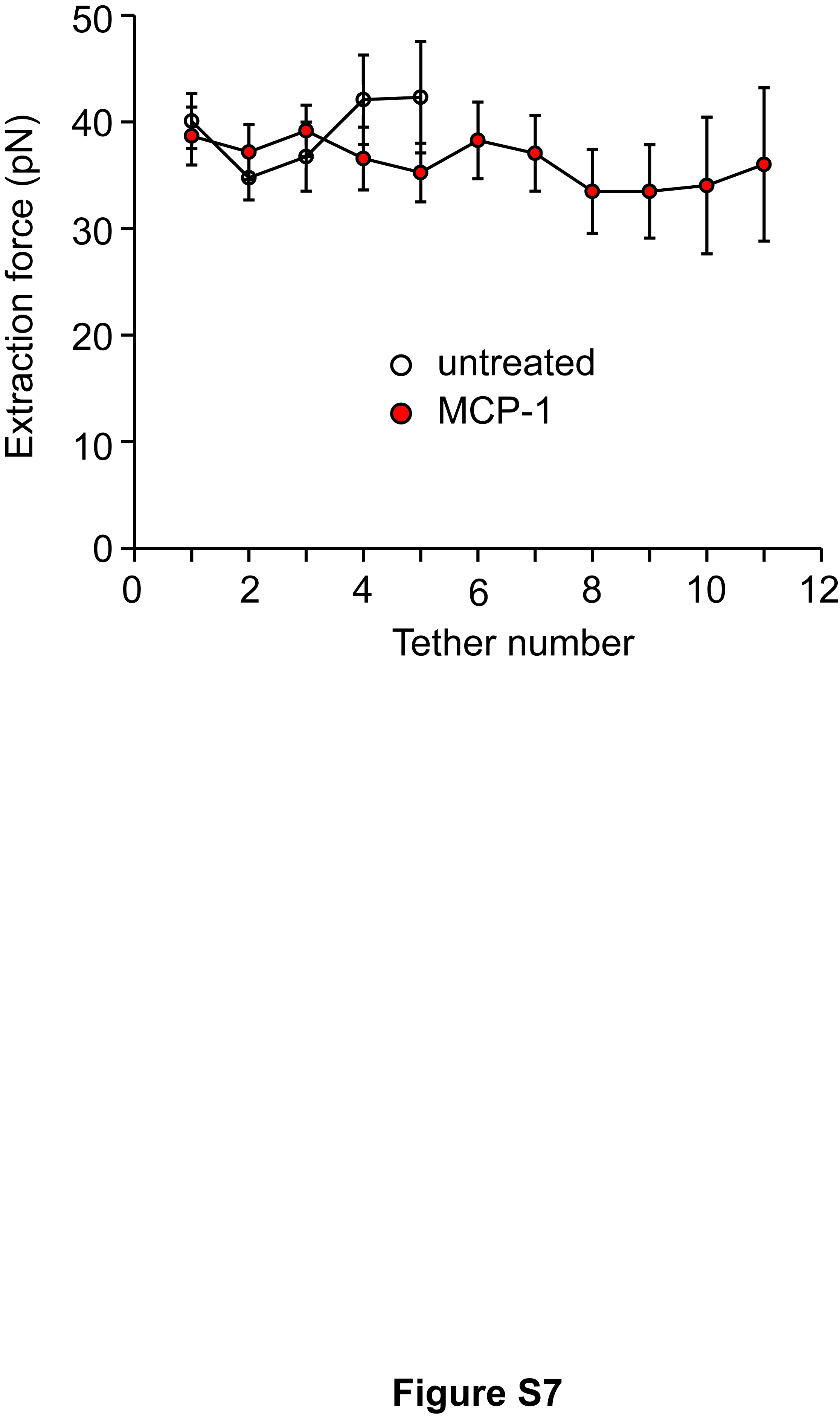

Supplement: Figure S7 — Quantification of individual tether extraction forces in whole cell adhesion curves. Each point represents the average tether extraction force for each observed tether in the whole cell adhesion curve. The tether number corresponds to the chronological occurrence of the tether. Error bars are s.e.m. A total of 61 force measurements from 10 cells were used to generate the untreated cell plot. 41 force measurements from 10 cells were used to generate the MCP-1-stimulated cell plot. (TIF) [file pone.0064187.s007.tif]
